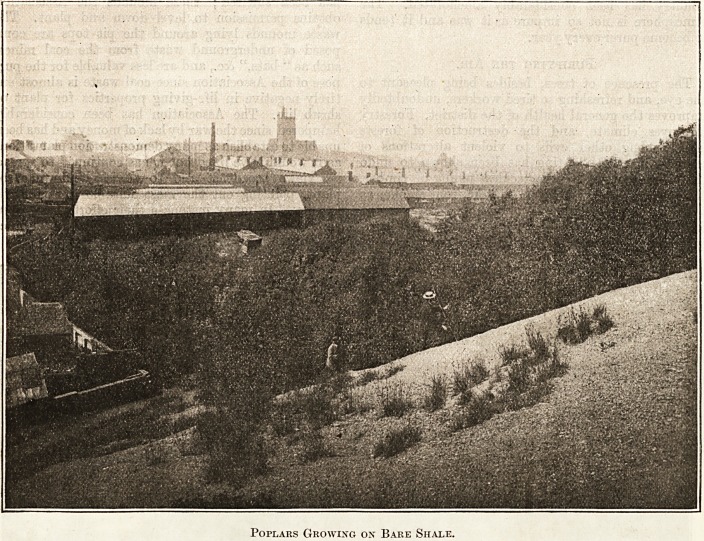# Replanting the Black Country

**Published:** 1923-04

**Authors:** 


### REPLANTING THE BLACK COUNTRY.

BY A RESIDENT OBSERVER.

THAT portion of Staffordshire and Worcestershire which is known throughout the kingdom as the Black Country, bids fair in the future to belie its title. For generations it has been regarded as an area of huge chimneys that by night and day belch forth smoke and flame, a country of ugliness, a desolate waste of pit and foundry rubbish heaps, the atmosphere of which, polluted by dense black smoke from a thousand stacks, is unfit to be breathed by man or beast. Many writers—Dickens, perhaps, the most memorable of them—have pictured it as an inferno where men stripped to the waist worked at huge furnaces till they could work no longer. Such is the reputation of the Black Country.

A Typical Pit Bank.

### Thirty Thousand Acres of Ruin.

In past ages the Black Country, in common with other parts of the kingdom, was to a large extent forest, and it is only since man commenced to utilise the coal under its surface, and to erect furnaces for the making of iron and steel and factories of every description that blackness has come upon it. Industrial activity has not only made the atmosphere murky, but has brought in its wake ruin to the once beautiful countryside until to-day there are some 30,000 acres of land used for the mere heaping of rubbish from the collieries and blast furnaces. For miles at a stretch nothing is to be seen but pit mounds and cinder banks. Spoil has ceased to be tipped upon it, and nature is busy clothing it with grasses and other humble vegetation. There is no denying that these banks are hideous and their ugliness is no doubt more striking to strangers than to those that have been born and bred among them. But even those who have been accustomed to them from childhood, and therefore notice them least, are very quick to see and to appreciate, when on a holiday trip, the refreshing beauty of wood and pasture in more favoured districts.

### Planting the Wastes.

But the Black Country is not fated to be for ever a desert. Efforts are being made in many quarters to restore some portion of their old beauty and usefulness to districts made desolate by the hand of man. It is not suggested that these neighbourhoods will ever become health resorts, but at least they can be so transformed as to be pleasing once again to the eye. Inspired by the great success which has attended the efforts at re-beautifying the similar country around Charleroi and other iron-making and coal-mining spots in Belgium, and at the same time providing new timber for their use, interested parties in this area set out by means of tree planting to see what could be done here. Public support was soon forthcoming and in 1903 the Midland Reafforesting Association was formed in Birmingham having as its object the replanting of waste grounds. Since that date trees have been set in plantations in many parts of the district and the Association has been busy giving expert advice on planting to private landowners who wished to improve their land, to the education authorities who have encouraged the establishing of plantations by school-children, and to the local authorities who have spent large sums of money in turning pit mounds into parks and recreation grounds. It was recognised that by improving the surroundings of the iron and steel workers and their children their health would be improved and undoubtedly the health of the Black Country has been considerably ameliorated. The atmosphere is not so impure as it was and it tends to become purer every year.

### Purifying the Air.

The presence of trees, besides being pleasant to the eye, and refreshing to tired workers, undoubtedly improves the general health of the district. Forestry improves climate, and the destruction of forests leads among other evils to violent alterations of temperature. Replanting has been shown to undo the harm wrought by the reckless felling of timber. Moreover, trees help to free the air from smoke, as a leaf picked from any tree and rubbed between the fingers will show. Woods are thus a natural purifying agent, and as such contribute directly toward improving the health of a district. To the activities of the Re-afforesting Association, then, some of the credit is due for the betterment of the health of the districts in which plantings have been carried out. The improvement is no doubt also due in some measure to the increasing use of power or producer gas for with its adoption is disappearing the bulk of the Black Country smoke. Even now much of it is preventable, and the Association is continually appealing to managers of industrial concerns to assist in reducing the volume of smoke. The best ground for re-afforesting in Staffordshire is the blast-furnace slag mound because of the iron in the earth left in the great cinders out of which the pig iron has been smelted; these mounds the Association obtains permission to level down and plant. The waste mounds lying around the pit tops are composed of underground waste from the coal mines, such as " bats," &c., and are less valuable for the purpose of the Association since coal waste is almost entirely negative in life-giving properties for plant or shrub life. The Association has been considerably hampered since the war by lack of money and has been unable to establish as many demonstration plantations as it would have liked but it has continued to spread its views broadcast and to advise landowners to plant, pointing out the most suitable trees for the locality. In many instances their efforts have been rewarded, and private plantations have been successfully established.

Poplars at Moorcroft, Moxley.

### The Creation of Sylvan Beauty.

So energetically has this work gone on that an inspection of the district shows numerous plantations of varying size, some already well wooded and a beauty to behold. The Association has in some places 20 years' growth of trees to show as a result of its labours. An excellent example of the work can be seen from the Great Western Railway. A new Isolation Hospital was started at Moorcroft, near Moxley, close to Wolverhampton, some years ago. Attached to it was a waste of forty-two acres, most of it genuine pit mound, but part was a sand pit and part was the site of the well-known Waterloo steep bank of slag supports thriving young willows, and an elder is growing in the old stokehole. At Wednesbury and Wolverhampton pit banks have been reclaimed, and to-day there are two parks recovered from waste which bid fair to become spots of real beauty. Already they are the playgrounds of hundreds of happy youngsters, who are watching the trees grow with themselves, and take a deep interest in the progress of these infant shrubs, some of which they have personally helped to plant. The Education Authority is taking a keen interest in the work of tree planting, and many school plantations have been started, while avenues of

Poplars Growing on Bare Shale.

furnace. This the Association planted in 50 plots by arrangement with the Joint Smallpox Hospital. To-day it is a beautiful colony, containing some 30 species of trees. There has also been afforestation at Reed's Wood, Walsall (where the trees are now well grown despite the close proximity of a chimney, discharging dense fumes), at the Birchills, Bentley, Pelsall, Wednesbury, West Bromwich, Wolverhampton, Dudley, Pensnett, Kingswinford, Cradley, and elsewhere. In most cases success is being achieved in spite of atmospheric disadvantage. At Dudley trees have grown with furnaces and forges reeking all round them. At Corngreaves, near Cradley, though oaks and conifers are feeble, birches are growing luxuriantly on a pit tip, which is still being added to and is actually on fire at one end. A healthy young trees are to be seen in most districts planted by the children themselves, many in remembrance of fathers, brothers, or other relatives killed in the Great War.

### Why Not Extend the Work ?

Everywhere in the Black Country miniature forests are springing up, and in all quarters trees are growing and thriving upon pit waste, upon furnace slag, and even upon the ash of burned out shale. Groves even of infant trees are better neighbours than unsightly dust heaps, and each tree that is planted becomes an incentive to plant more. The correct planting of the trees is a scientific matter, and great care is exercised in this particular. Otherwise much useless expenditure might be incurred.

## Figures and Tables

**Figure f1:**
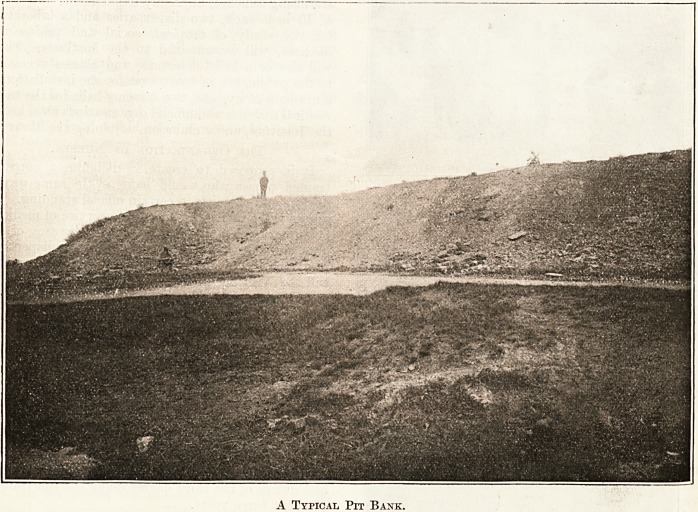


**Figure f2:**
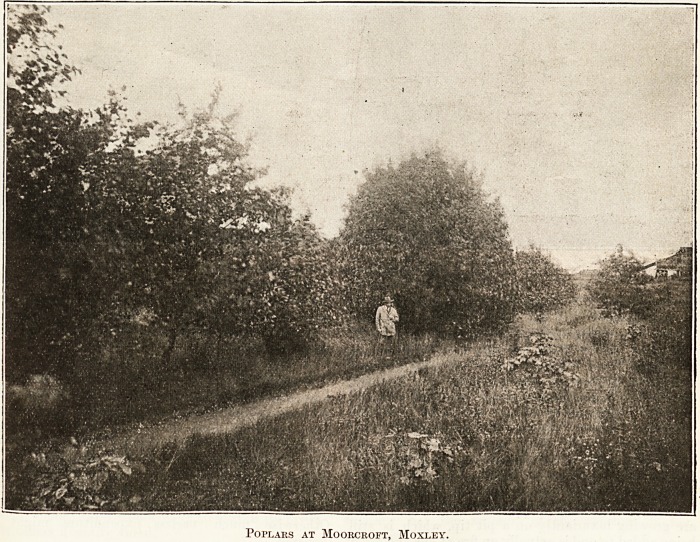


**Figure f3:**